# Variation in DNA Substitution Rates among Lineages Erroneously Inferred from Simulated Clock-Like Data

**DOI:** 10.1371/journal.pone.0009649

**Published:** 2010-03-11

**Authors:** Rachel S. Schwartz, Rachel Lockridge Mueller

**Affiliations:** Department of Biology, Colorado State University, Fort Collins, Colorado, United States of America; Field Museum of Natural History, United States of America

## Abstract

**Background:**

The observation of variation in substitution rates among lineages has led to (1) a general rejection of the molecular clock model, and (2) the suggestion that a number of biological characteristics of organisms can cause rate variation. Accurate estimates of rate variation, and thus accurate inferences regarding the causes of rate variation, depend on accurate estimates of substitution rates. However, theory suggests that even when the substitution process is clock-like, variable numbers of substitutions can occur among lineages because the substitution process is stochastic. Furthermore, substitution rates along lineages can be misestimated, particularly when multiple substitutions occur at some sites. Although these potential causes of error in rate estimation are well understood in theory, such error has not been examined in detail; consequently, empirical studies that estimate rate variation among lineages have been unable to determine whether their results could be impacted by estimation error.

**Methodology/Principal Findings:**

To evaluate the extent to which error in rate estimation could erroneously suggest rate variation among lineages, we examined rate variation estimated for datasets simulated under a molecular clock on trees with equal and variable branch lengths. Thus, any apparent rate variation in these datasets reflects error in rate estimation rather than true differences in the underlying substitution process. We observed substantial rate variation among lineages in our simulations; however, we did not observe rate variation when average substitution rates were compared between different clades.

**Conclusions/Significance:**

Our results confirm previous theoretical work suggesting that observations of among lineage rate variation in empirical data may be due to the stochastic substitution process and error in the estimation of substitution rates, rather than true differences in the underlying substitution process among lineages. However, conclusions regarding rate variation drawn from rates averaged across multiple branches are likely due to real, systematic variation in rates between groups.

## Introduction

There is significant interest in estimating rates of gene evolution [e.g. 1], [2], [3], [4], [5] and differences in such rates among species, clades, and over time [e.g. 6], [7], [8]. Early estimates of substitution rates assumed a molecular clock (i.e. a constant rate of evolution) [9], and protein sequence data initially supported this hypothesis [see review by 10]. However, it has since been suggested that genes rarely evolve according to a clock model, with significant variation in substitution rates even among closely related species [e.g. 6], [11], [12].

Across species, rate variation in orthologous genes has been attributed to differences in the biology of different organisms. For example, species with shorter generation times may have higher substitution rates because more rounds of DNA replication in germs cells produce more mutations per unit time [13], [14], [15]. Species with higher metabolic rates may have higher substitution rates due to the potential relationship between DNA damage and mutation rate [16]. Constraints on sequence function can lead to lower substitution rates, whereas positive selection can lead to higher substitution rates [17]. Rate variation between paralogous genes has been attributed to differences in selection pressures among gene copies with different functions [6], [18], [19], [20].

Before suggesting a link between biological processes and variation in substitution rates, it is necessary to determine whether rate variation occurs in a dataset. Methods of measuring rate variation fall into two broad categories: (1) descriptive statistics of rate variation among lineages, clades, or other subsets of the tree, and (2) statistical tests for deviation from a molecular clock. Descriptive statistics of rate variation include the overall range of variation and the standard deviation of rates across the tree [e.g. 7], [11], [21]. Statistical tests for deviation from a molecular clock in part or all of the tree include relative rate tests [22], [23], likelihood ratio tests [24], and comparisons between average rates for clades [e.g. 2] or paralogous genes [e.g. 6]. Relative rate tests evaluate whether the difference between one species or clade and an outgroup is significantly different from the expectation derived from the difference between another species or clade and the outgroup [25]. Likelihood ratio tests [26] evaluate whether a phylogenetic tree estimated without enforcing a molecular clock is significantly more likely than a tree estimated when a clock is enforced [e.g. 6]. Given an observation of variation in substitution rates, it is then possible to examine correlations between rate and measures of biological processes [e.g. 27], differences in rates between two clades or paralogous genes [e.g. 6], differences in rates between a single lineage and the rest of a clade [e.g. 1], or differences between contemporary and ancestral substitution rates [e.g. 20]. The presence of rate variation can also suggest the need for more complex models in phylogenetics and divergence dating [see review by 28].

All measures of rate variation, and all inferences linking rate variation to some aspect of organismal biology, depend on accurate estimates of substitution rates. The independent process of substitution at different sites results in the expectation that, when the substitution process is clock-like, the number of substitutions on a branch will be drawn from a Poisson distribution [29]. This process leads to differences between the actual and expected numbers of substitutions in limited datasets, and consequently, estimated rates that differ from the true rate. Thus, given finite data, the stochastic substitution process can mislead researchers by erroneously suggesting variation in the underlying biological substitution process among different lineages [10].

Another challenge associated with rate estimation is the underestimation of the number of substitutions along some branches, as a result of multiple substitutions at some sites, particularly as phylogenetic distance (i.e. branch length) increases [29], [30]. When branches are of equal length stochastic variation in the number of uncounted substitutions can lead to different estimates of the length of each branch, erroneously suggesting rate variation. When branches are of unequal length, an erroneous estimate of rate variation can result from greater underestimation of substitution rates on longer branches [30]. This problem has been also been described as the node-density effect: clades with greater taxon sampling have shorter branches (estimated accurately), while clades with more sparse taxon sampling have longer branches (underestimated), resulting in a higher estimated substitution rate for the former [31], [32]. Substitution models are intended to correct these problems [33]; however, because no model summarizes the substitution process perfectly, and because models can be estimated inaccurately [30], any correction for multiple substitutions will also be imperfect [34]. Thus, both stochastic variation in the substitution process and error in rate estimation can have significant impacts on overall estimates of rate variation among lineages, even when the underlying biological substitution process is constant. In this study, we use the general term “error in rate estimation” to include both variation in estimated rates due to the stochastic substitution process, and rate misestimation due to uncounted substitutions.

Although these potential sources of error in rate estimation have been described previously, their cumulative effects on estimates of rate variation among lineages have not been examined in detail. Thus, studies that link substitution rate variation to particular biological processes have not been able to consider whether error in rate variation estimation affects their conclusions. In this study, we simulated datasets under a molecular clock and estimated (1) substitution rates for each lineage, and (2) substitution rate variation among lineages. Thus, any rate variation we observe reflects error in rate estimation, including both stochastic variation due to the Poisson process, and incorrect estimates of the number of substitutions along lineages; our analyses allow us to differentiate between these two sources of error and avoid confounding error with true variation in the substitution process. We used datasets of both equal and unequal branch lengths simulated using both simple and more realistic substitution models (based on the results of Mueller [21] for plethodontid salamanders). We observed rate variation among lineages in these simulations; thus, observations of among-lineage rate variation in empirical data may be due, in part, to error in rate estimation, rather than true variation in the underlying biological substitution process. Finally, we suggest cases for which erroneously estimated rate variation may and may not affect conclusions based on such variation.

## Results

### Bayesian-estimated variation in rates across all branches for 8-taxon trees

**Figure 1:**
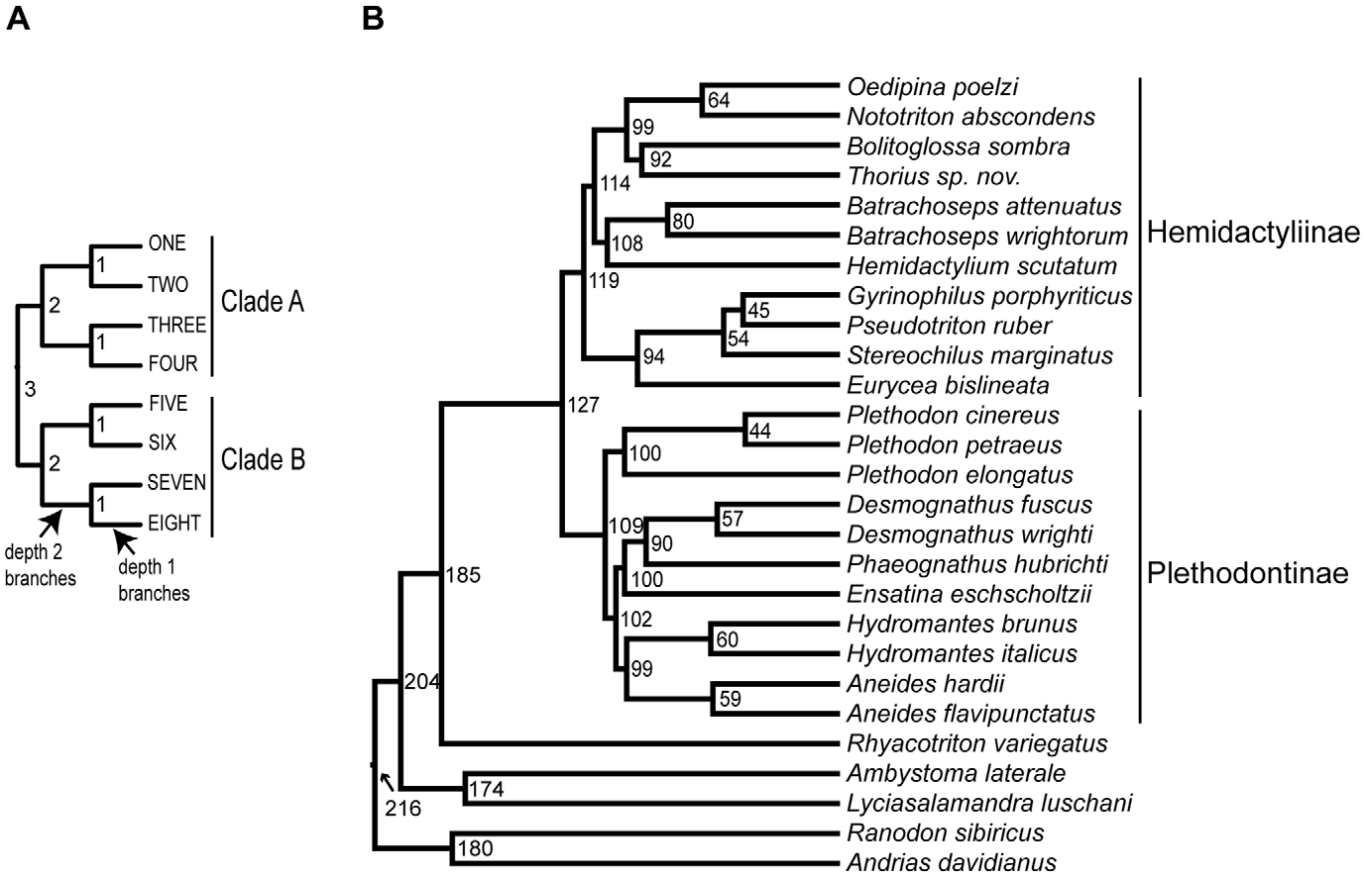
Trees used for data simulation. (a) Eight-taxon, ultrametric trees used to simulate data with a molecular clock enforced. Node ages are labeled in millions of years. Branch lengths for simulations (in substitutions/site) were obtained by multiplying the age of the branch by 11 different rates. (b) 27-taxon trees based on the tree topology for plethodontid salamanders from Mueller et al. [35], with dates from Mueller [21] in millions of years. Branch lengths for simulations (in substitutions/site) were obtained by multiplying the average rate estimated by Mueller [21] for each of five mitochondrial genes by the length of the branch in years.

**Figure 2:**
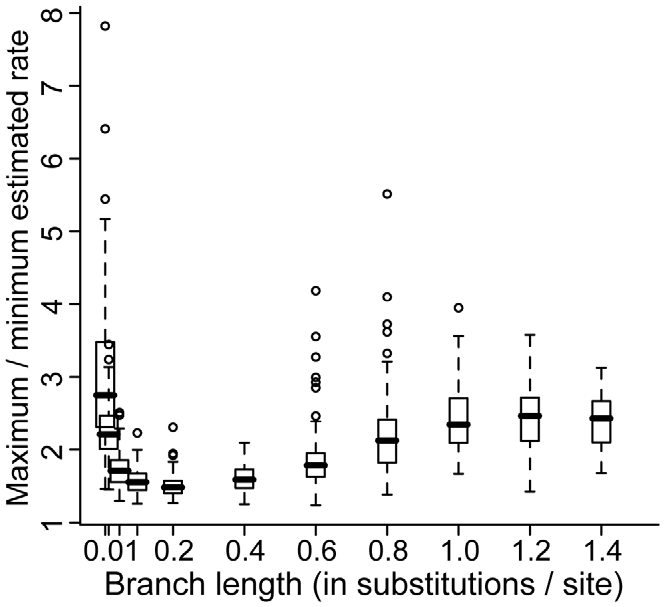
Rate variation observed for datasets simulated on 8-taxon trees and analyzed in a Bayesian framework. Data were simulated with a molecular clock model.

We first evaluated rate variation estimates for datasets simulated on 8-taxon trees with equal-length branches ranging from 0.01 to 1.4 substitutions/site and rates estimated in a Bayesian framework. Rate variation was measured as maximum/minimum estimated rate; to evaluate the causes of observed rate variation, we compared this result to the variation expected due to the stochastic substitution process. We observed >2-fold variation in substitution rates in the majority of datasets of the shortest and longest branch lengths (Figures 1 and 2; Table 1). For longer branch lengths, the variation in substitution rates was also significantly more than predicted from the stochastic substitution process (Table 1). With the exception of the shortest branch length, variation in estimated substitution rates increased as branch length increased (Figure 2). For each set of simulations, the mean estimated rate was the same for each dataset (ANOVA P>0.34 for all trees).

**Table 1:** Descriptive statistics of estimated rate variation for 8-taxon equal-branch-length trees.

Branch length	0.01	0.02	0.05	0.1	0.2	0.4	0.6	0.8	1.0	1.2	1.4
Max. est. fold variation (Bayesian)	7.8	3.4	2.5	2.2	2.3	2.1	4.2	5.5	4.0	3.9	3.1
Max. est. fold variation (ML)	15	3.7	2.6	2.3	2.4	2.2	7.5	19.2	3472041	302065	257835
# of datasets with est. fold variation >2 (Bayesian)	87	75	13	1	1	5	21	58	86	80	82
# of datasets with est. fold variation >2 (ML)	93	81	16	2	1	8	38	89	97	100	100
Poisson expectation of fold variation	4.4	2.6	1.8	1.5	1.3	1.2	1.2	1.2	1.1	1.1	1.1
# of datasets with est. fold variation > expected (Bayesian)	11	16	34	60	92	100	100	100	100	100	100
# of datasets with est. fold variation > expected (ML)	19	26	36	61	92	100	100	100	100	100	100
# of datasets for which the clock was rejected (Bayesian)	0	2	0	2	2	0	2	7	0	8	0
# of datasets for which the clock was rejected (ML)	0	0	0	0	0	0	0	0	0	0	0
Max. est. fold variation between depths (Bayesian)	1.7	1.4	1.3	1.2	1.2	1.2	1.3	1.6	1.7	1.8	1.8
Max. est. fold variation between depths (ML)	1.8	1.5	1.3	1.2	1.2	1.2	1.2	1.4	1.9	67	119
Max. est. fold variation between clades (Bayesian)	1.9	1.4	1.2	1.2	1.2	1.2	1.2	1.3	1.22	1.2	1.3
Max. est. fold variation between clades (ML)	2.0	1.5	1.2	1.2	1.1	1.1	1.2	1.3	1.27	39.8	77.6

Overall rate variation, rate variation between depths, and rate variation between clades, were calculated from rates estimated in Bayesian and Maximum Likelihood (ML) frameworks for 100 simulated datasets for trees with branch lengths of 0.01 – 1.4 substitutions/site. Estimated rate variation expected due to the Poisson process was calculated as (μ+2σ)/(μ−2σ). Each dataset was tested for rejection of the molecular clock using Bayes factor comparisons for Bayesian analyses with and without the clock enforced, and a likelihood ratio test for ML analyses.

Because a common method of determining whether the substitution process is clock-like is to compare results with and without the molecular clock enforced, we also tested whether the molecular clock could be rejected by any of our datasets. The molecular clock was rejected for very few datasets (Table 1). Additionally, we tested whether estimated substitution rates were normally distributed around the mean as expected from the Poisson process. Normality was rejected for the distribution of estimated substitution rates for just 2–9 datasets per set of 100 datasets.

### ML-estimated variation in rates across all branches for 8-taxon trees

**Figure 3:**
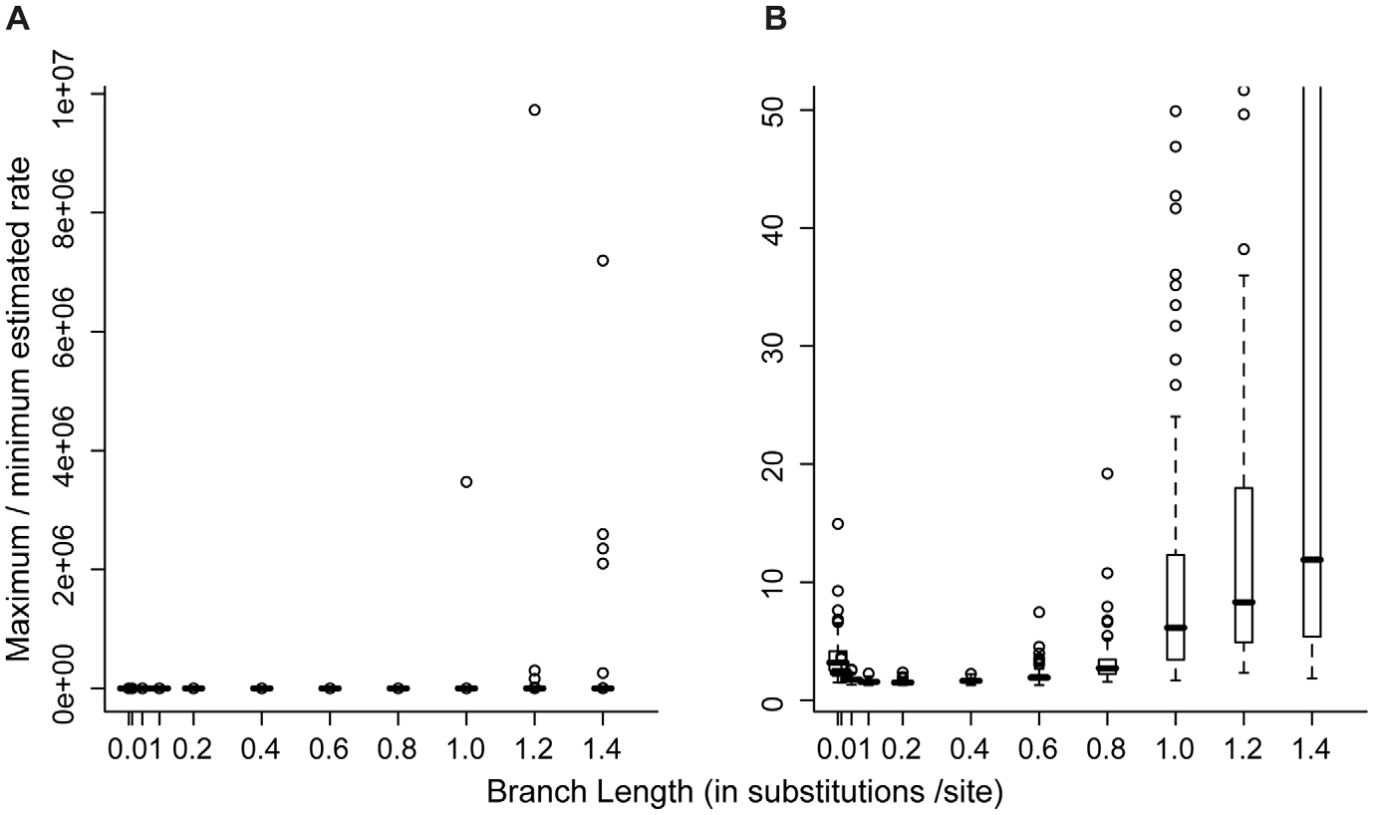
Rate variation observed for datasets simulated on 8-taxon trees and analyzed in an ML framework. Data were simulated with a molecular clock model. (a) rate variation including all data; (b) rate variation excluding results for some datasets to show the range of rate variation for shorter branch lengths.

When rates were estimated in an ML framework we observed >2-fold variation in substitution rates for the majority of datasets of the shortest and longest branch lengths (Figure 3; Table 1). In calculating estimated rate variation, branch length estimates of 0 substitutions/site were removed from analyses because they would result in rate variation of infinity; thus, our estimates of rate variation underrepresent the total range of such variation. With the exception of simulations of branch lengths of 1.2 substitutions/site (ANOVA P = 0.002), the mean estimated rate was the same for each dataset (ANOVA P>0.35). The molecular clock was not rejected in any case. A normal distribution was rejected for estimated substitution rates for 5–10 datasets out of 100 for branch lengths of 0.01–1.2 substitutions/site, and 32 datasets for branch lengths of 1.4 substitutions/site.

### Rate comparisons between depths and between clades for 8-taxon trees

Averaging estimated rates across multiple branches resulted in less erroneous estimates of rate variation. The difference between average Bayesian-estimated rates for different groups (i.e. clades or depths) was <2-fold for all simulated datasets (see Table 1 for more details). The difference between average ML-estimated rates for different clades was <2-fold for all simulated datasets (see Table 1 for more details). The difference between average ML-estimated rates for different depths was also <2-fold for simulated datasets of branch lengths of ≤1 substitution/site; however, although it was not significant in any case, the difference in averaged estimated rates was considerably higher for longer branch lengths (Table 1).

### Variation in estimated rates on the 27-taxon variable-branch-length tree

**Figure 4:**
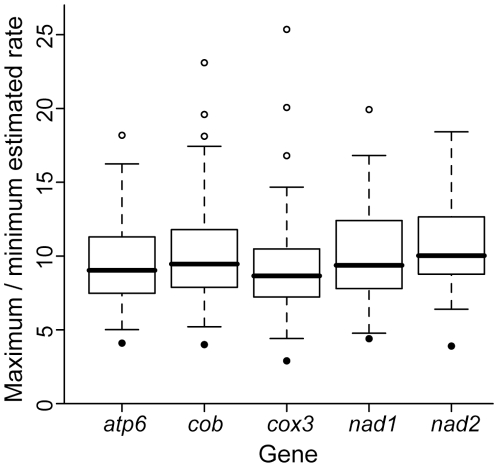
Rate variation observed for datasets simulated on 27-taxon trees and analyzed in a Bayesian framework. Data were simulated with a molecular clock model using the model and model parameters estimated for five mitochondrial genes for plethodontid salamanders [21] (boxes). For comparison, rate variation observed for empirical data from plethodontid salamanders from Mueller [21] is also shown (filled circles).

**Figure 5:**
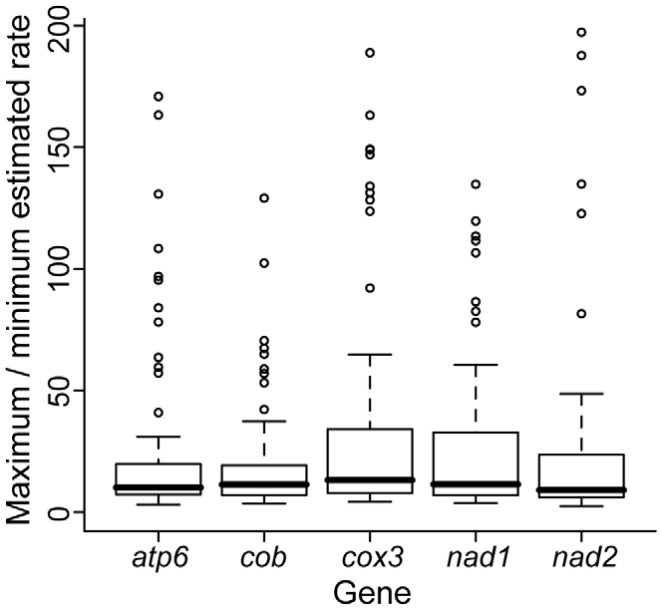
Rate variation observed for datasets simulated on 27-taxon trees and analyzed in an ML framework. Data were simulated with a molecular clock model using the model and model parameters estimated for five mitochondrial genes for plethodontid salamanders [21] (boxes). Up to five outliers were omitted for each gene in order to view the center of the distribution more clearly. Variation was ≤763-fold for *atp6*, ≤510-fold for *cob*, ≤777-fold for *cox3*, ≤257-fold for *nad1*, and ≤723-fold for *nad2*.

Trees with unequal-length branches more closely approximate empirical datasets; thus, we simulated datasets on the 27-taxon plethodontid salamander phylogeny of Mueller et al. [35] using parameters for five mitochondrial genes with branch lengths set using the average rate for each gene and estimated divergence dates [21]. All five genes simulated under these clock-like conditions showed surprisingly high estimated rate variation among the 27 lineages for both Bayesian and ML analyses (Figures 4 and 5). Despite these differences, molecular clock tests did not reject clock-like evolution for any ML analyses. In a Bayesian framework, the estimated marginal likelihood was significantly higher for clock-like model in nearly all cases, and not significantly different for the remaining cases. There was no difference between the two major clades in estimated average rates (<1.3 fold difference for Bayesian analyses and <1.5-fold for ML analyses).

Because we estimated substantial rate variation among lineages using simulated clock-like data based on parameters from empirical plethodontid data, we compared these results to the rate variation estimated from the original empirical data [21] to test whether such empirical rate variation could reflect error. The variation in rates estimated for each gene for the empirical dataset analyzed in a Bayesian framework [21] was, in all cases, less than the erroneous variation in rate estimated for comparable clock-like datasets (Figure 4). Additionally, estimated rates were not significantly different between the two major plethodontid clades in the empirical dataset (<1.2 fold difference).

## Discussion

### Error in estimated rate variation and potential impacts of such error

We observed substantial variation in estimated rates of substitution, both in Bayesian and ML frameworks, when considering all branches across trees simulated under an enforced molecular clock. Simulations were conducted using a clock-like substitution process; therefore, this observed rate variation must be due to error in rate estimation, both as a result of the stochastic substitution process producing different numbers of substitutions than expected along lineages, and misestimation of rates due to uncounted substitutions. This variation provides an example of the potential for error in estimates of rate variation for empirical datasets. Levels of rate variation observed for datasets simulated with realistic parameters were even greater than those for simple 8-taxon simulations, suggesting that the level of error in simple simulations provides a minimum for potential error in empirical datasets.

Estimates of rate variation derived from empirical data in plethodontid salamanders were less than those derived from comparable datasets simulated under an enforced molecular clock, suggesting that the empirically-estimated rate variation may reflect error rather than true differences in the underlying biological substitution process. In addition to the stochastic variation and rate misestimation error that are the focus of this study, the node-density effect can cause error in variation [32]. We tested for this effect in plethodontids by regressing total path length (i.e. total inferred substitutions from root to tip) against the number of speciation events for each gene. However, the number of nodes did not explain a significant portion of the estimated rates, and thus, would not have contributed to estimated rate variation; this likely reflects the fact that most branch lengths were outside the range affected by systematic underestimation [30]. This result suggests that the types of error we discuss in this study, rather than the node density effect, explain the estimated rate variation in the plethodontid dataset.

Our results suggest that some conclusions of other studies based on apparent variation in substitution rates may be based, at least in part, on error in rate estimation. For example, inferences based on the estimated rate of a single lineage, such as an exceptionally high rate [e.g. 1], may be based on error in rate estimation on that lineage. Additionally, correlations observed between substitution rate and variation in substitution rate [e.g. 21] may reflect greater error in rate estimation for faster-evolving genes (Figures 2 and 3). Error in rate estimation may also obscure actual correlations that exist between substitution rate and variation in particular traits. For example, Thomas et al. [36] found no evidence of a correlation between body size and substitution rate in invertebrates; such negative results may, in fact, reflect rate estimation error obscuring biological signal in the data. Furthermore, the general consensus that the molecular clock is an overly simple model of molecular evolution, based on the observation that most datasets exhibit variation in substitution rates across lineages, may be in part based on error in rate estimation such as we observed in this study.

Error in estimating rate variation can significantly impact phylogeny and divergence date estimation; thus, the observation of rate variation in many datasets has led to the development of substitution models attempting to accommodate such rate variation [37], [38], [39], [40]. However, if these models are fitting error rather than true differences in the underlying substitution process, then their use may introduce, rather than reduce, error in phylogeny estimation. Even when the topology is estimated correctly, divergence dates are likely to be affected by error in rate estimation, particularly if only a few calibration points are used. Our results suggest the potential for substantial error in substitution rate estimates for individual branches; thus, if the rate on a branch used for calibration is estimated incorrectly, this error will be propagated over the whole tree. This source of error compounds several other difficulties inherent in divergence date estimation. For example, the combination of systematic error in the number of substitutions estimated on long branches with calibrations located on long branches can lead to the underestimation of substitution rates and, therefore, the overestimation of dates on shallower nodes [41]. Furthermore, some error is inevitably associated with fossil calibrations, despite more realistic analytical techniques that incorporate fossil dates as probability distributions rather than point estimates of divergence times [42], [43].

Previous observations of rate variation among lineages resulted in the suggestion that divergence dates among clades could be estimated accurately by using mean substitution rates for multiple species within each clade [44]. This divergence dating approach would also minimize potential impacts of the types of error we discuss in this study. Rate-smoothing methods such as those implemented in PATHd8 and r8s may also reduce the effects of error by autocorrelating rates on neighboring branches [45], [46], [47].

Our observations of rate variation across branches, despite a clock-like substitution process in simulations, are consistent with previously suggested sources of error in rate estimation. The rate variation we observed for simple datasets simulated on phylogenies with shorter branches was likely due to the probabilistic substitution process, which generates a set of branch lengths that follow a Poisson distribution, even when the rate of the underlying substitution process is constant. However, the difference between the actual and expected number of substitutions produced by the probabilistic substitution process does not account for the estimated rate variation for phylogenies with longer branches. As branch length increases, the effect of the standard deviation in the Poisson distribution decreases in comparison to the mean when considering expected rate variation. Thus, we expect rate variation estimated as a result of the stochastic substitution process to decrease as branch lengths increase. In contrast, our results show an increase in mean rate variation as branch length increases (Figure 2) and we attribute this to methodological error in branch length estimation. Both of these sources of error, combined with different levels of systematic error in rate estimation on branches of different lengths, likely led to erroneous estimates of rate variation in datasets of variable branch lengths (Figure 4).

In simulations, the substitution process conforms to a specified model; thus, the imprecise fit of the model to the data that can cause rate estimation error for empirical data [34] should not apply here. Our results suggest that even analyses using accurate models can yield imperfect estimates of multiple substitutions. In empirical studies, additional error in rate variation estimation likely results from using models that only approximate the substitution process.

### Correct estimation of variation in substitution rates

In contrast to rate variation estimates across all branches of a tree, averaged rates across clades or branch depths showed little difference in estimated rates in either Bayesian or ML frameworks for the majority of branch lengths. Thus, error in estimates of rates on individual branches was not sufficient to mislead comparisons of rates among clades or among different tree depths in simulations. This lack of difference in rates results partly from the Poisson distribution of the actual number of substitutions around the expected number; when multiple samples (i.e. rates estimated from different branches) from a single distribution are averaged, no rate variation is expected. It also suggests that methodological error in rate estimation is equally distributed around the mean estimated rate. Similarly, we observed little difference in estimated rates between the two major clades of plethodontid salamanders when using empirical data. These similar rates between clades are also consistent with the possibility, suggested by the comparison of rate variation across all branches for empirical and simulated data, that observed rate variation in this dataset reflects error in rate estimation.

These results suggest that conclusions based on average rates across multiple lineages are unlikely to be affected by error in rate estimation. Thus, comparisons of average substitution rates between clades suggesting a faster rate for one clade are likely accurate [e.g. 2], [15], [48]. For example, our results would not affect the observed link between rates of molecular evolution and life history in flowering plants, based on multiple comparisons between average rates for sister clades of different life history types [15]. Similarly, comparisons of rates for paralogous genes would be correct; thus, conclusions regarding rates for sub- or neofunctionalized genes [e.g. 18], [19] would not be affected by this source of error. Correlations between substitution rates in different lineages and variation in traits (such as those related to life history) are also likely unaffected by our results. For example, in a study correlating substitution rate with body mass and longevity [11], error in rate estimation would have functioned as random noise in the data; because it is not systematic, such error would not have altered the conclusions. Similarly, Davies and Savolainen [49] found correlated rates of change for molecular and morphological characters, a result that is also unlikely to have been affected by error. Finally, patterns across multiple genes, even for a single lineage, are also unlikely to be affected by our results; the error we observed is random and would not be consistent across loci.

Because of the substantial variation in substitution rates we observed in our data, we might have expected to reject a molecular clock for each dataset; this was not the case. However, as noted by Li [50], the failure to reject the molecular clock cannot be taken as evidence that the substitution process is clock-like. Furthermore, the sizes of our datasets fall within the range for which clock deviation tests are known to be overly conservative; thus, even rate variation due to differences in the biological substitution process at these levels may not result in rejection of the molecular clock [51].

### Conclusion

In summary, we suggest that estimates of among-lineage rate variation are prone to error. However, the impacts of such error on conclusions drawn from empirical datasets depend on the measure of rate variation used in analysis. We suggest that conclusions based on (1) the overall range of rate variation across all branches in a tree, and/or (2) rates estimated on individual branches should be drawn with caution. However, because error in rate estimation produces normally distributed rate estimates, conclusions based on rates averaged across multiple branches are likely not affected.

## Materials and Methods

We used two types of simulations to test the extent to which error in substitution rate estimation can lead to overall error in rate variation estimation: (1) simple trees of equal-length branches, and (2) more realistic trees of mixed-length branches. Using trees with equal-length branches avoided the confounding factor of different levels of multiple substitutions per site expected when comparing long and short branches. However, trees with mixed-length branches are more similar to those that might be observed in empirical datasets. In both cases, there was no rate variation in the simulation process and the tree was ultrametric. By using a simulation approach, error in rate estimation can be isolated from the confounding problem of error in substitution model estimation present in empirical datasets.

Branch lengths for each dataset were estimated in both Bayesian (using MrBayes [52]) and ML (using PAUP* [53]) frameworks with the model specified to match the one used for simulations and model parameters (detailed below) estimated from the data. Bayesian analyses were run for one million generations with two runs of four chains each; trees were sampled every 100 generations, and 3000 trees were discarded as burnin when summarizing results.

### Variation in substitution rates for simple trees with equal-length branches

The first set of datasets was simulated on 8-taxon balanced trees with equal-length branches of 0.01, 0.02, 0.05, 0.1, 0.2, 0.4, 0.6, 0.8, 1.0, 1.2, and 1.4 substitutions/site (Figure 1a). One hundred datasets of 1 kb each were simulated in SeqGen [54] for each tree using an HKY model with equal base frequencies and a transition/transversion ratio of two. The rate on each branch was calculated by fixing the ages of each depth 1 node at one million years and each depth 2 node at two million years (Figure 1a). Thus, for each of the 1100 datasets (11 branch lengths×100 simulations each), we obtained a distribution of 13 rate estimates (one for each branch) in both Bayesian and ML statistical frameworks. If the substitution process draws from a Poisson distribution, 95% of estimated rates are expected to be within two standard deviations (2σ) of the mean (μ); when this is not the case, we can attribute some error in rate estimation to the estimation process itself.

For each simulated dataset, rate variation was summarized in three different ways: (1) across all branches (i.e. the maximum estimated branch length divided by the minimum estimated branch length); (2) between branch depths (i.e. average estimated branch length for depth 2 branches divided by average estimated branch length for depth 1 branches (Figure 1a)); and (3) between clades (i.e. average rate for all branches in clade A divided by average rate for all branches in clade B (Figure 1a)). Comparisons of rates across all branches provide a general summary of the overall rate variation in empirical datasets, while the latter two comparisons are often used to suggest systematic differences in substitution rates due to differences in selective regimes between groups or over time.

One of our goals was to distinguish erroneously estimated rate variation resulting from the stochastic substitution process from that resulting from misestimation of substitution rates. We did this in the following way, based on characteristics of the Poisson distribution: for each set of simple simulations, 100 datasets with 13 branches each yielded in an expectation of 65 branches with estimated rates not within two standard deviations of the mean. If such branches are equally distributed among datasets, at least 35 datasets should have estimated rate variation less than (μ+2σ)/(μ−2σ); when this is not the case, we infer that error in rate estimation is present and causing more extensive rate variation than expected based on the stochastic substitution process alone.

The significance of the difference in rates between clades or depths was determined based on whether error bars of one standard deviation overlapped. Because a common method of determining whether the substitution process is clock-like is to compare results with and without the molecular clock enforced, we also tested whether the molecular clock could be rejected by any of our datasets. We conducted these tests using (1) Bayes factor comparisons for Bayesian analyses with and without the clock enforced (a Bayes factor >10 was considered significant) [55], and (2) a likelihood ratio test with a chi-square statistic for ML analyses [56].

To further describe the distribution of rates estimated for each dataset, we tested each of the 2200 distributions for normality [57]. We examined the distributions of estimated rates to determine whether our three descriptions of rate variation would be disproportionately affected by outliers, or whether such descriptions would summarize sets of normally distributed rates. A normal distribution also ensures that parametric statistics are appropriate. We used ANOVA to test whether the mean estimated rates for each of the 100 datasets for each branch length were equal. We note the non-independence of lengths estimated for branches on a single tree; however, there are no currently equivalent tests that allow non-independent samples.

### Variation in substitution rates estimated on a mixed-branch-length tree

The second set of datasets was simulated on a more realistic tree of 27 taxa based on the plethodontid salamander phylogeny of Mueller et al. [35] (Figure 1b). Trees used for simulations were again clock-like and ultrametric, with branch lengths calculated using the average rates of substitution for five mitochondrial genes (*atp6*, *cob*, *cox3*, *nad1*, and *nad2*) (0.445, 0.617, 0.640, 0.426, and 0.366 substitutions/site respectively) and the divergence dates estimated by Mueller [21] (Figure 1b). One hundred datasets for each of the five trees were simulated in SeqGen using parameters estimated for each gene [21]. The true tree topology was imposed as a constraint in analyses. Substitution rates on each branch for each simulated dataset were calculated by fixing the node dates on the estimated trees to be equal to those used for simulations. Variation in estimated substitution rates was calculated across all branches and between the two major plethodontid clades (i.e. average scaled branch length for all branches within Hemidactyliinae divided by average scaled branch length for all branches within Plethodontinae) (Figure 1b). The significance of the difference in rates between clades was determined based on whether error bars of one standard deviation overlapped. We did not compare rates for different depths on the tree because this is not feasible for unbalanced trees. As with 8-taxon simulated datasets, we tested whether the molecular clock could be rejected using Bayes factors and the likelihood ratio test.

Our analyses of apparent rate variation in clock-like datasets provide a measure of error that might exist in estimates of rate variation from empirical data. More specifically, if rate variation for empirical data falls within the rate variation erroneously estimated for comparable clock-like data, this would suggest that the empirical rate variation is due to error in rate estimation rather than true differences among lineages in the underlying biological substitution process. Mueller [21] estimated rate variation across 27 plethodontid lineages for each of the 15 mitochondrial genes and concatenated tRNA genes. In the current study, we simulated datasets based on parameters estimated for five of these genes, but enforcing a molecular clock. The observed rate variation across all branches for empirical data (calculated using a Bayesian approach) for these five genes in plethodontid salamanders was 4.1-fold for *atp6*; 4-fold for *cob*; 2.9-fold for *cox3*; 4.4-fold for *nad1*; and 3.9-fold for *nad2* [21]; we compared such rate variation to rate variation estimated from our comparable clock-like simulations. Additionally, we compared rates between major plethodontid clades to determine whether variation between clades was greater than expected based on the clock-like simulations.
